# Bamboo sheath—A modified branch based on the anatomical observations

**DOI:** 10.1038/s41598-017-16470-7

**Published:** 2017-11-23

**Authors:** Shuguang Wang

**Affiliations:** Southwest Forestry University, Kunming, 540000 China

## Abstract

Culm sheath has always been considered a modified leaf. In this study, the anatomical structure of the culm sheath from *Fargesia yunnanensis* was analyzed to determine whether it originated from the foliage leaf blade or from the branch. The vascular bundles of the culm sheath showed greater similarity to the branches in shape and anatomical structure. In contrast to foliage blades, there are no midribs in culm sheaths. Stomatal density in the culm sheath is greater in the adaxial than it is in the abaxial epidermis, which is the opposite of that found in foliage leaf blades, and that density shows greater similarity to branches than it does to foliage blades. Fusoid cells are distributed on both sites of the vascular bundles in foliage blades, whereas culm sheaths have, instead, a few parenchyma cells that disintegrate and form air cavities when the sheath matures. Additionally, the culm sheath has no bulliform cells or trichome in its epidermis, and the shape of its long cells shows greater similarity to those of branches. Therefore, culm sheath is a modified branch, rather than a modified leaf.

## Introduction

Sheath is an important morphological characteristic in Poaceae plants. In bamboo plants, there are three types of sheaths: the culm sheath (shoot sheath), the rhizome sheath and the leaf sheath. The culm sheath has a key role in systematic bamboo classification, and Chatterjee and Raizada^1^ have shown that the morphology of culm sheaths is species specific and can be used to identify bamboos to the species level. Culm sheaths can be green or another colour, but they are usually persistent to some degree, even after turning brown, and can remain on mature culms for some time, depending on their species-specific nature^2,3^.

In young shoots, every node bears a sheath, which embraces the developing internode distal to its insertion point^3^. The sheaths envelope the culm’s most fragile section, helping to prevent possible damage by providing crucial stiffness^4–6^. To date, most studies have focused primarily on the mechanical functions of culm sheaths. Singh *et al*.^7^ established that the sheath completely surrounds and protects new shoots. Wong^2^ determined that culm sheaths have a protective role, encasing the tender lower part of a bamboo internode. The contribution of sheaths to the entire rigidity of the culms in *Arundinaria tecta* reached 33% on average^5^. In *Arundo donax* and *Miscanthus* species, research has shown that sheaths provide significant mechanical properties necessary for the development of culms^8,9^. Kempe *et al*.^10^ further verified the mechanical role of the leaf sheath is essential for culm stability during development and growth.

**Figure 1:**
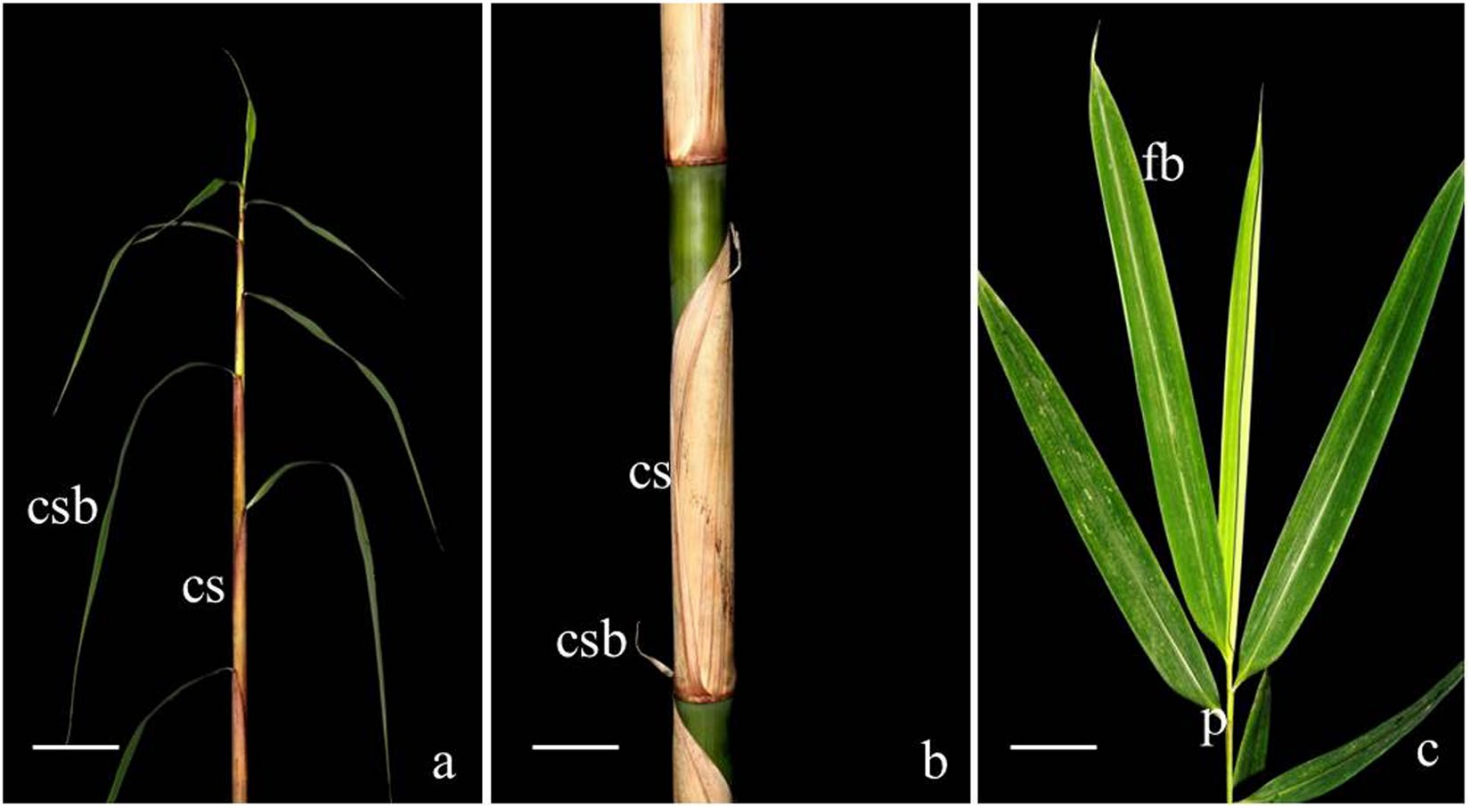
Culm sheath and foliage leaf of *Fargesia yunnanensis*. cs, culm sheath; csb, culm sheath blade; flb, foliage leaf blade; fls, foliage leaf sheath; p, petiole. (**a**) Culm sheath at the top of bamboo culms, showing the leaf-like sheath blade, which is much longer than the foliage leaf blade. Bar = 10 cm. (**b**) Culm sheath at the middle of bamboo culms, showing the small sheath blade. Bar = 10 cm. (**c**) Foliage leaf blade and foliage leaf sheath joining, which is the petiole. Bar = 1 cm.

Although the emerging shoots of different bamboo species vary widely in colour, shape and size, all culm sheaths are composed of the sheath proper and the sheath blade^2,3^. Auricles, ligules and oral setae may or may not be present. For example, the experimental materials used in the present study came from *Fargesia yunnanensis*, which has no ligules or auricles at the top of culm sheath (Fig. 1a,b). The culm sheath blade varies greatly in size, position and morphology^3^. As shown in Fig. 1, the sheath blades at the base of the culm are quite small, whereas, at the top of the culms, they are leaf-like and even longer than the foliage leaf blades. In addition, the culm sheath shape is very similar to that of the leaf sheath, and both have the same morphological characteristics. Wong^2^ determined that all the leaf-like structures, including the rhizome sheath, culm sheath and branch sheath were homologous. It is because of their peculiar leaf-like shape that bamboo culm sheaths are considered modified leaves. However, foliage leaves have a leaf “stalk” joining the blade to the sheath (Fig. 1c), which is usually called the *petiole*, but the bamboo culm sheath does not have that organ. The petiole is considered an important distinction between bamboos and other grasses. Because there is a petiole between the foliage leaf blade and the leaf sheath, we considered whether the leaf sheath more likely originated from a branch rather than from a modified leaf.

In this study, the anatomical characteristics of bamboo culm sheaths, sheath blades and foliage blades were observed and analyzed to draw distinguish them and to determine whether the culm sheath is a modified leaf or originated from a branch.

## Results

### Anatomical characteristics of different organs

**Figure 2:**
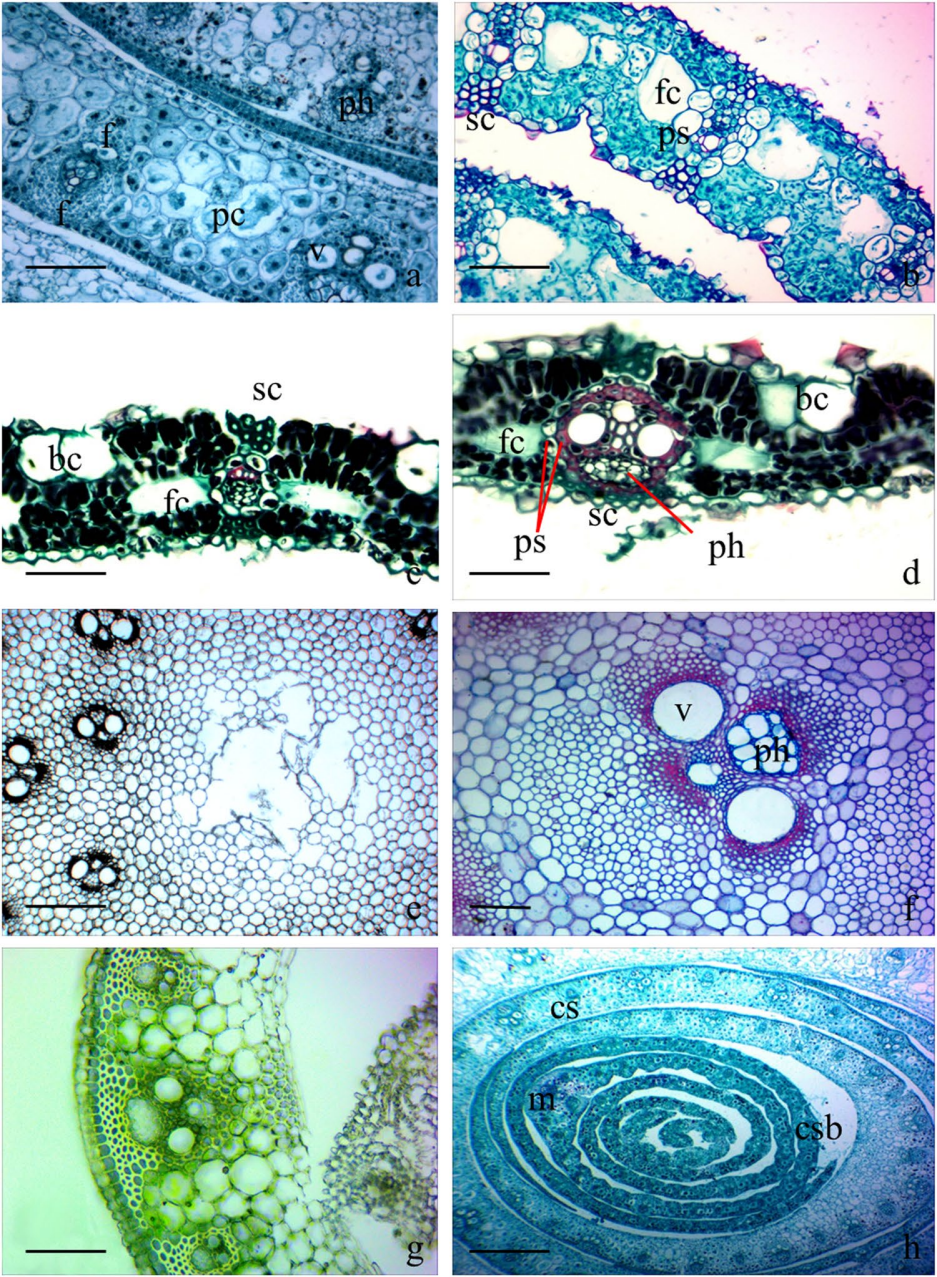
Cross sections of culm sheath, culm sheath blade, foliage leaf blade, leaf sheath, branch and shoot culm. bc, bulliform cells; bs, bundle sheath; cs, culm sheath; csb, culm sheath blade; f, fibre cells; fc, fusoid cells; ph, phloem; ps, parenchymatous sheath cells; sc, sclerenchyma cell; v, vessel element. (**a**) Anatomical structure of culm sheaths showing vascular bundles surrounded by fibers and large parenchyma cells between the vascular bundles. Bar = 20 μm. (**b**) Anatomical structure of culm sheath blades showing vascular bundles surrounded by one layer of large parenchymatous sheath cells, with sclerenchyma cells on the adaxial and abaxial sites of vascular bundles. Few bulliform cells can be seen on the adaxial epidermis, and significant transparent fusoid cells are shown. Bar = 20 μm. (**c**) Anatomical structure of foliage leaf blades showing bulliform cells on the adaxial epidermis, vascular bundles with only one large layer of parenchymatous sheath cells and fusoid cells on both sites of vascular bundles. Sclerenchyma cells were also observed on the adaxial and abaxial sites of vascular bundles. Bar = 20 μm. (**d**) Anatomical structure of foliage leaf blades showing vascular bundles with two layers of bundle sheath cells, one layer of parenchymatous sheath cells and one layer of sclerenchyma sheath cells. Bar = 20 μm. (**e**) Anatomical structure of branches. Bar = 60 μm. (**f**) Vascular bundles in shoot culms. Bar = 20 μm. (**g**) Hand section of the leaf sheath. Bar = 20 μm. (**h**) Cross section of culm sheaths and sheath blades at the tops of shoots, showing the significant difference between them. Midrib and fusoid cells could be observed in sheath blades but not in culm sheaths. Bar = 60 μm.

The vascular bundles in the culm sheath are composed of vessel elements and phloem and are surrounded by many fibre cells (Fig. 2a). Especially in the protoxylem vessels and phloem, the fibres comprise a fibre cap, which separately forms into a phloem fibre sheath or a metaxylem fibre sheath. This anatomical structure is similar to that of the vascular bundles in branches and culms (Fig. 2e,f). The vascular bundles in the foliage leaf sheath show similar anatomical characteristic (Fig. 2g). However, in culm sheath blades, there is only one type of vascular bundles, and those vascular bundles are surrounded by a single layer of parenchymatous sheath cells (Fig. 2b), instead of fibre cells. In addition, sclerenchyma cells were observed at the adaxial and abaxial sites of each vascular bundle in the culm sheath blades. In foliage leaf blades, vascular bundles showed similar anatomical characteristics (Fig. 2c,d), i.e. the sclerenchyma cells were located at adaxial and abaxial sites of vascular bundles, and one or two layers of sheath cells surrounded the vascular bundles. Bulliform cells are easily observed in the abaxial epidermis of the foliage leaf blade, but they do not appear in sheath blades, which might be due to the short time the culm sheath blade survives during the rapid growth of bamboo shoots, making it unnecessary for the culm to develop bulliform cells during that short life circle.

In general, the anatomical structure of the vascular bundles in the culm sheath differed significantly from those in the culm sheath blades and in the foliage leaf blades, but they were the same as those in branches and shoot culms. All the phloem in the vascular bundles of foliage leaf blades, sheaths, branches or culms was located towards the abaxial epidermis. There was, however, no midrib in the culm sheath, whereas it could clearly be observed in the foliage blades (Fig. 1h), which was also a significant difference between the culm sheaths and the leaf blades.

### Fusoid cells and distribution of starch granules

**Figure 3:**
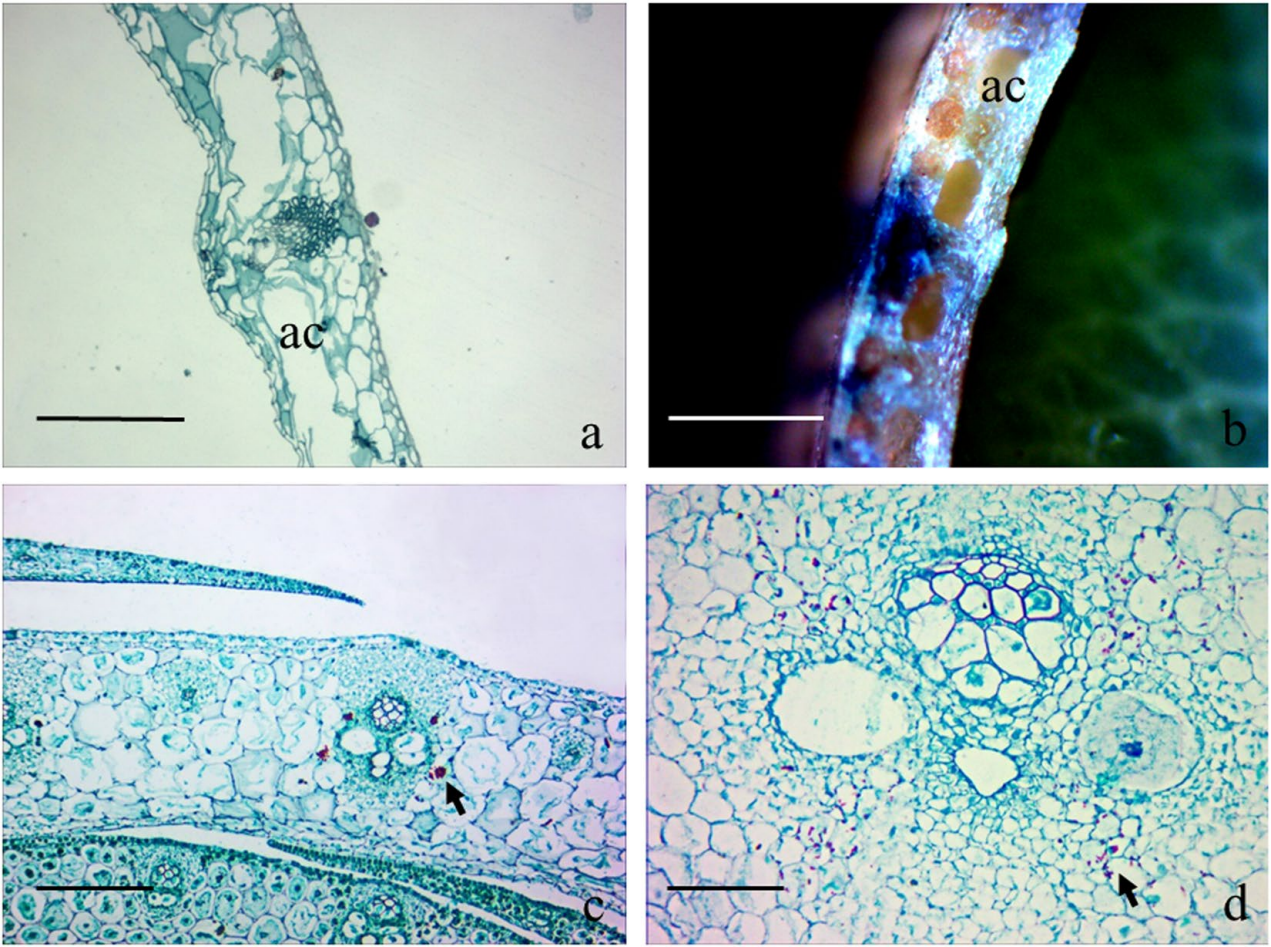
Air cavities in culm sheath, and starch granules stained in the culm sheath and young shoot culms. ac, air cavities. (**a**) Parenchyma cells disintegrated into air cavities in culm sheaths. Bar = 80 μm. (**b**) Air cavities observed under anatomical lens. Bar = 100 μm. (**c**) Starch granules localized in culm sheath (arrow). Bar = 20 μm. (**d**) Starch granules localized in young shoot culms (arrow). Bar = 20 μm.

Fusoid cells are present on both sides of all vascular bundles in the culm sheath blades (Fig. 2g) and the foliage blades (Fig. 2c,d), and they are easily distinguished by their extremely thin walls and by being achlorophyllous and transparent. However, few fusoid cells, except for a few large parenchyma cells, could be observed between the vascular bundles of culm and leaf sheaths (Fig. 2a,g). When the culm sheaths ceased their growth and dropped off, those huge parenchyma cells on both lateral sides of the vascular bundles began to disintegrate (Fig. 3a) and formed air cavities (Fig. 3b), which lead to an anatomical structure that appeared similar to those of foliage blades. Therefore, the air cavities formed from a few parenchyma cells were also the important distinguishing characteristic for the culm sheath.

According to the localization of starch granules, the starch granule distribution in the culm sheath is very similar to that in the shoot culms (Fig. 3c,d), which might imply that they use similar sugar transportation and storage methods.

### Stomata distribution in different organs

**Figure 4:**
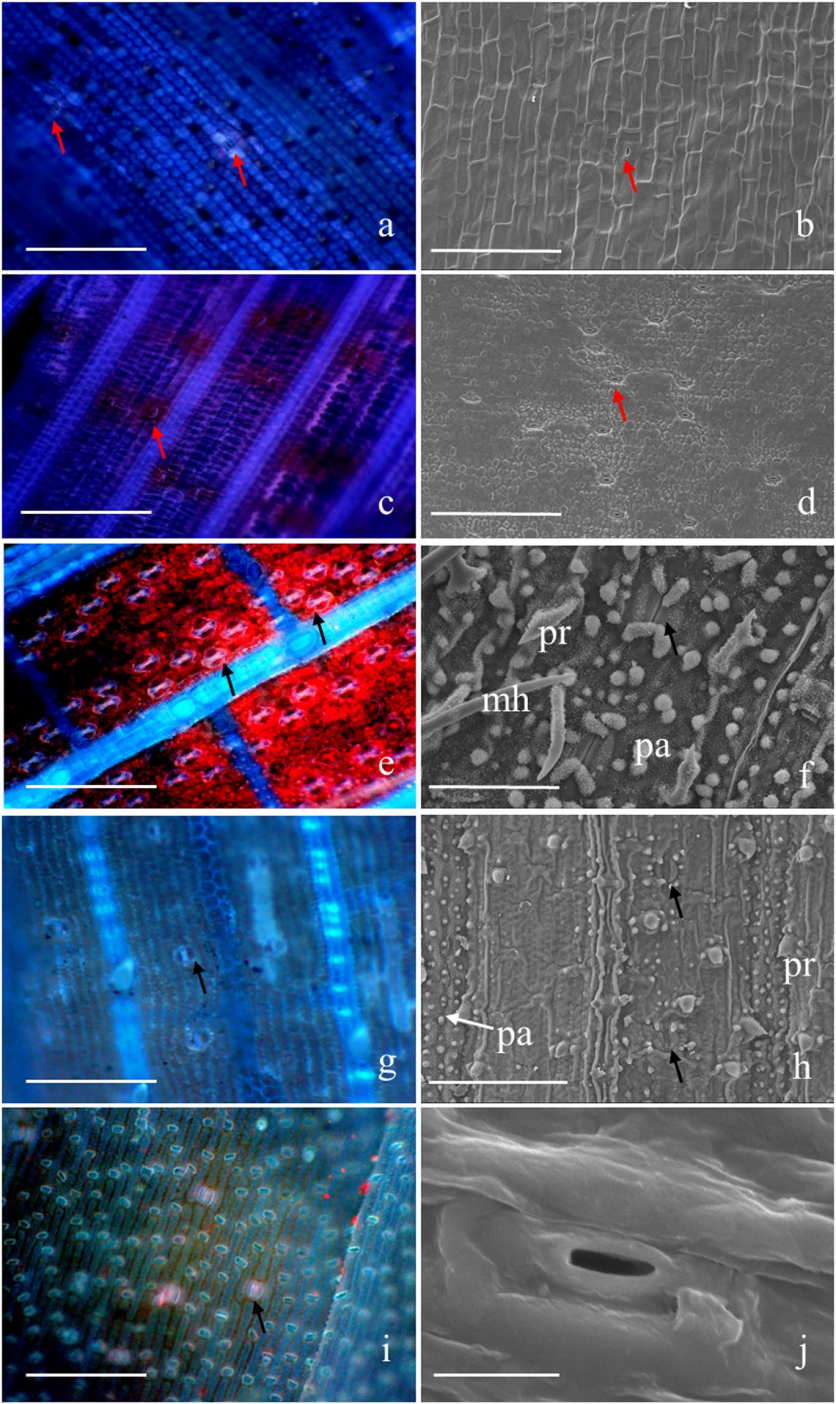
Stomata in culm sheath, foliage leaf blades and branches, indicated by arrows. pr, prickle; pa, papilla; mh, microhair. (**a**) The abaxial epidermis of culm sheath under fluorescence microscopy, showing a few stomata. Bar = 200 μm. (**b**) The abaxial epidermis of the culm sheath under SEM. Bar = 200 μm. (**c**) The adaxial epidermis of culm sheath under fluorescence microscopy, showing more stomata. Bar = 200 μm. (**d**) The adaxial epidermis of culm sheath under SEM. Bar = 200 μm. (**e**) The abaxial epidermis of foliage blade under fluorescence microscope, showing the large number of stomata. Bar = 100 μm. (**f**) The abaxial epidermis of foliage blade under SEM. Bar = 50 μm. (**g**) Observations of stomata in the adaxial epidermis of foliage blade under fluorescence microscope, showing fewer stomata than that found in the abaxial epidermis. Bar = 200 μm. (**h**) The stomata in the adaxial epidermis of the foliage blade under SEM. Bar = 100 μm.

**Figure 5:**
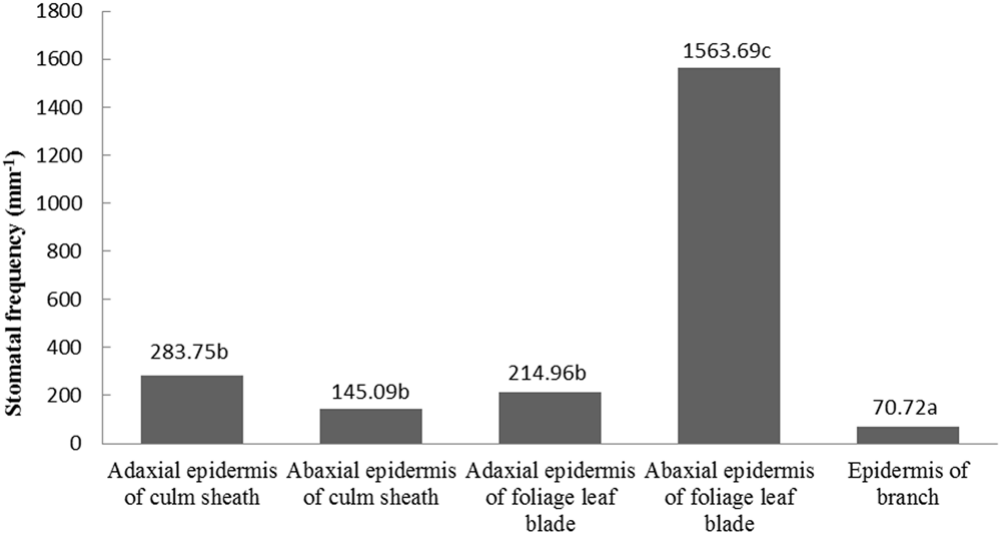
Stomatal density in various organs of *Fargesia yunnanensis*.

Viewed with a fluorescence microscope and a scanning electron microscope (SEM), more stomata were found in the adaxial epidermis of the culm sheath from *F*. *yunnanensis* (Fig. 4c,d), reaching a density of 283.75 mm^−2^ (Fig. 5), than were found in the abaxial epidermis (Fig. 4a,b), which had a stomatal density of 145.09 mm^−2^. Although more stomata was observed in the abaxial epidermis (1563.69 mm^−2^) than in the adaxial epidermis (214.96 mm^−2^) of the foliage leaf blades (Figs 4e–h and 5), that distribution was just the opposite in the culm sheath. Branches also had a few stomata observed in their culm skin, which had a stomatal density of just 70.72 mm^−2^ (Figs 4i,j and 5). By comparison, the stomatal density of culm sheaths was far less than that of the foliage leaf blades; therefore, the culm sheath was more closely aligned with the branch in stomatal density, although the density difference between them was also significant.

Upon SEM review, the stomata were open in the culm sheaths and in the epidermis of the branches (Fig. 4b,d,j), whereas they were closed in the foliage leaf blades (Fig. 4f,h), which might be due to their differences in stoma structure. In addition, the shape of the abaxial epidermis cells of the culm sheath showed greater similarity to that of the branches (Fig. 4a,i), and were significantly different from that of the foliage leaf blades (Fig. 4g) when viewed with a fluorescence microscope. The lateral walls of the long cells were deeply sinuous in the foliage leaf blades when compared with those of the culm sheaths and branches. In addition, numerous prickles, microhairs and papillae were observed on the abaxial and adaxial epidermis of the foliage leaf blade (Fig. 4f,h) but were absent on the epidermis of both culm sheath and branch. The stomata of the foliage leaf blade were usually covered by four papillae in the abaxial epidermis (Fig. 4f) but were not found in the epidermis of the culm sheath or branch (Fig. 4b,d,j).

## Discussion

In bamboo plants, the leaves can be divided into two forms with different functions. One form is the culm sheath, which encloses much of an internode and has a protective role for the tender lower part of the internode when its tissues are actively dividing and lengthening. The other form is the foliage leaf on the finer branches for photosynthesis^2^. Each form bears a blade at the top of sheath. Venation of the leaf blade is parallel, and there are three types of veins: the midrib, the secondary veins and the tertiary veins. The culm sheath comprises many vascular bundles, which run longitudinally parallel. Liese and Köhl^3^ considered that culm sheaths to be modified leaves and to have the same parts as leaves, but the parts are differently proportioned. Foliage leaves have a petiole joining the leaf sheath and the blade, which does not exist in culm sheaths. In addition, there is no midrib in the culm sheath, but both the sheath blade and the foliage leaf blade have midribs, based on observations of the transverse sections of culm sheath.

The anatomical structure of bamboo foliage leaf blades is similar to that of other grasses, with bulliform cells and large vascular bundles with characteristic bundle sheaths^11^. Although few bulliform cells could be observed in the sheath blade or culm sheath proper, so the bulliform cells also became to be the significant difference between culm sheath and foliage blade. Shields^12^ demonstrated that bulliform cells become flaccid, allowing the plant to bend or infold to reduce leaf transpiration surface during periods of excessive water losses. Apparently, it was unnecessary for culm sheaths to develop bulliform cells because they mainly function to protect and to provide stiffness for the fresh culms. In the foliage blades of Poaceae, one- or two-layer bundle sheaths may envelop the conducting tissues^13^. When both present, the outer sheath is called *endodermis* and contains starch or chloroplast, whereas the inner sheath is the *pericycle* and consists of sclerenchymatic cells^13,14^. Many grasses have two cell layers surrounding their vascular bundles, whereas others have only a single layer^15^. Wang *et al*.^16^ determined there were two types of vascular bundles in *F*. *yunnanensis*, and all cells of the outer and inner bundle sheaths formed a cytoplasmic continuum by the plasmodesmata. However, this study found only one type of vascular bundle in the culm sheath proper, which was significantly different from that of the foliage leaf blade in shape. Unlike those in foliage blades, the vascular bundles in culm sheaths are surrounded by neither endodermis nor pericycle, but are instead surrounded by isolated xylem or phloem fibre sheath, which is also the case for bamboo culms, in which the fibres occur as fibre caps (sheaths) surrounding the conducting elements^17^.

Vieira *et al*.^13^ determined that sclerenchymatic sheath extensions could be observed in all bundles and that they reach one epidermis or both. Sclerenchyma cells are observed at both adaxial and abaxial sites in the vascular bundles and reach both epidermis layers in *F*. *yunnanensis* foliage blades, but they are not found in culm sheath proper, which is a significant anatomical difference between foliage leaf blades and the culm sheath proper.

Foliage leaf blades also have other appendages that were significantly different from the culm sheath proper, including fusoid cells, stomata, bulliform cells and prickles and papillae on the epidermis. Fusoid cells are an important character in defining the bambusoid type of a leaf’s anatomy^18^. March and Clark^19^ considered fusoid cells to be a prominent feature of the leaves of bamboos and their early divergence from grasses. Wang *et al*.^16^ also reported that fusoid cells originated from large parenchyma cell, as shown in the nuclei. In the present study, the fusoid cells were easily observed in foliage leaf blades and culm sheath blades but were absent in the culm sheath proper, which, instead, had some parenchyma cells between the vascular bundles. In the mature culm sheath, those parenchyma cells break down and form air cavities close to the veins, which look similar to the foliage leaf blade in shape and anatomical structure, which might be a phenomenon of convergent evolution.

Most bamboos have their stomata on the abaxial surface of their foliage leaf blades^20^. In contrast, more stomata are found on adaxial than the abaxial epidermis in the culm sheath proper. The quantity of stomata in the culm sheath is also closer to that of the branch and far less than that of the foliage leaf blade. In addition, there are numerous prickles and papillae in the foliage leaf that are absent in the culm sheath. The stomata of the foliage leaf blade are closed and are covered by four papillae, whereas, in the culm sheath proper and the branch, they are open and have no papillae. Therefore, the stomata are not as easily observed because the papillae grow over the stomatal cells in foliage leaf blades^21^. Terrel and Wergin^22^ suggested that the stomata may be protected by papillae.

Ellis^23^ defined bulliform cells as an intrinsic part of the epidermis, and Shields^12^ suggested bulliform cells served to prevent water loss from foliage blades by reducing the blade’s transpiration surface. However, bulliform cells do not exist within the culm sheath, implying their functional difference. Additionally, previous studies have shown that the long cells of foliage blades have sinuous walls^13^. However, in this study, the lateral wall of the long cells in the culm sheath was slightly undulated and shared greater similarity to that of branches but was significantly different from that of foliage leaf blades, with their deeply sinuous lateral walls. In addition, bamboo nodal buds are enclosed by the culm sheath proper, which is very similar to dicotyledons, in which axillary buds are hidden beneath fleshy leaf petioles.

## Conclusion

In this study, it was concluded that culm sheath was not a modified leaf blade but was, instead, a modified branch based on the following seven anatomical structures.

### Vascular bundles

In the foliage leaf blade, there are two types of vascular bundles, and each vascular bundle is surrounded by a one- or two-layer sheath, including one layer of large parenchymatous sheath cells. In culm sheath proper there is only a single type of vascular bundle, that bundle shows greater similarity with branches and culms in anatomical characteristics. In addition, the foliage leaf blades and culm sheath blades have a midrib, whereas the culm sheath does not.

### Sclerenchyma cells

In foliage leaf blade, sclerenchyma cells are distributed on both abaxial and adaxial sites in the vascular bundles and reach both layers of the epidermis. However, in culm sheath, there are no sclerenchyma cells between epidermis and the vascular bundle.

### Stomata

Culm sheath more closely resemble branches than foliage leaf blades in stomatal density. In the foliage leaf blade, the stomatal density is greater along the abaxial epidermis than it is along the adaxial epidermis, whereas the density is the opposite in the culm sheath proper. The stomata are open in both culm sheaths and branches, whereas they are closed in foliage leaf blades when viewed by SEM.

### Fusoid cells

Fusoid cells can be seen on both sites of vascular bundles in the foliage leaf blade, whereas they do not exist in the culm sheath.

### Bulliform cells

Bulliform cells are observed in foliage leaf blade but are not observed in the culm sheath.

### Long cells

The shape of the long cells in the culm sheath show greater similarity with branches than they do with foliage leaf blades.

### Trichome

In the foliage leaf blade, there are numerous prickles, papillae and microhairs on the epidermis, and the stomata are covered by papillae and are not easy seen. However, in the culm sheath proper, there are few trichomes found.

## Materials and Methods

### Plant materials

The bamboo came from the garden of the Southwest Forestry University (Yunnan Province, China) in October 2016. The culm sheath and culm sheath blades were cut from 1-month-old shoots of *Fargesia yunnanensis*, and the foliage leaf blades, foliage leaf sheaths and branches were from the mature bamboo germinated the prior year. Young shoots were also brought to the laboratory as experimental materials for anatomical comparisons.

### Paraffin sections

The fresh foliage leaves, culm sheaths, sheath blades and bamboo shoots were fixed directly in FAA (45% alcohol, 0.25% acetic acid and 1.85% formaldehyde) and dehydrated in a graded series of alcohol (began at 50%). Transverse sections (7 μm) were cut with a rotary microtome, stained with 1% alcoholic Safranin O (Sigma S-2255) in 50% ethanol, dehydrated in a graded series of ethanol and stained with Fast Green FCF (Ameresco 0689) (Fast Green, 1 g; clove oil, 100 mL; and 100% ethanol). The sections were mounted in Canada balsam.

In addition, some sections of culm sheath and bamboo shoots were stained with periodic acid–Schiff to determine the distribution of starch granules. After soaked in 0.5% KIO_4_ for 10 minutes, paraffin sections were soaked in Schiff’s reagent solution for 30 min, dehydrated in a graded series of ethanol and stained with Fast Green FCF.

### Hand sections

Hand sections were cut from fresh foliage leaf sheaths and culm sheath with a double-edged razor blade and then flattened in water. Samples were observed with a converted fluorescence microscope (Nikon E400) for the anatomical comparisons.

### Sliding sections

After preservation in a mixture of 50% alcohol, 10% glycerin and 40% water for about 1 month, the branches were cut to 17-μm thickness with a sliding microtome (Leica). Transverse sections were then double stained with 1% alcoholic Safranin O (Sigma S-2255) in 50% ethanol and Fast Green FCF and dehydrated in a graded series of ethanol. The sections were permanently mounted in Canada balsam.

### Microscopy

All paraffin and slide sections were observed with a video camera linked to a fluorescence microscope (Nikon E400) and a Lenovo computer. In addition, stomata were counted, and their distribution density was calculated with an SEM (Hitachi S-3000N) and a fluorescence microscope.

Before the observation with the SEM, all specimens were fixed in FAA, dehydrated in a graded series of alcohol and cut into sections by hand.

Before the observation with the fluorescence microscope, all sections were directly cut from fresh specimens. The culm sheath, culm sheath blade and foliage leaf blade were cut into 1 cm × 1 cm samples and placed on glass slides with a single drop of distilled water. For branches, the skin was peeled, and the stoma was observed by SEM and fluorescence microscope.

### Statistical analyses

Statistical analyses were performed using SPSS 13.0 software. Least significant difference was employed to analyze the differences in the number of stomata among the various specimens.
